# The burden attributable to headache disorders in India: estimates from a community-based study in Karnataka State

**DOI:** 10.1186/s10194-015-0574-9

**Published:** 2015-11-09

**Authors:** Girish N. Rao, Girish B. Kulkarni, Gopalkrishna Gururaj, Lars J. Stovner, Timothy J. Steiner

**Affiliations:** Department of Epidemiology, National Institute of Mental Health and Neuro Sciences (NIMHANS), Bangalore, India; Department of Neurology, National Institute of Mental Health and Neuro Sciences (NIMHANS), Bangalore, India; Department of Neuroscience, Norwegian University of Science and Technology, Edvard Griegs Gate, NO-7491, Trondheim, Norway; Norwegian Advisory Unit on Headaches, Nevrosenteret Øst, St Olavs University Hospital, Trondheim, Norway; Division of Brain Sciences, Imperial College London, London, UK

**Keywords:** Headache disorders, Migraine, Tension-type headache, Medication-overuse headache, Burden, Health policy, Willingness to pay, India, South-East Asia, Global campaign against headache

## Abstract

**Background:**

Headache disorders are common worldwide, causing pain and disability. India appears to have a very high prevalence of migraine, and of other headache disorders in line with global averages. Our objective was to estimate the burdens attributable to these disorders in order to inform health policy.

**Methods:**

In a door-to-door survey, biologically unrelated adults (18–65 years) were randomly sampled from urban and rural areas of Bangalore and interviewed by trained researchers. The validated structured questionnaire enquired into several aspects of burden.

**Results:**

Of 2,329 participants (non-participation rate 7.4 %), 1,488 (63.9 %; 621 male, 867 female) reported headache in the preceding year. Symptom burden was high. Migraine (1-year prevalence 25.2 %) occurred on average on 28 days/year but, in 38.0 % of cases (*ie*, 9.6 % of adults), on ≥3 days/month (≥10 % of days). All causes of headache on ≥15 days/month (prevalence 3.0 %) occurred on a mean of 245 days/year. Both these and migraine were rated severe in intensity.

Participants with headache lost 4.3 % of productive time; those with migraine lost 5.8 % (equating to 1.5 % from the adult population). Lost paid worktime accounted for 40 % of this, probably detracting directly from GDP.

We estimated population-level disability attributable to migraine using the disability weight from GBD2010 for the ictal state (0.433). Mean disability per person with migraine was 1.8 %, reducing the functional capacity of the entire adult population by 0.46 %.

Fewer than one quarter of participants with headache had engaged with health-care services for headache in the last year. Actual expenditure on headache care was greatest among those with headache on ≥15 days/month (especially probable medication-overuse headache), but otherwise not high. Expressed willingness to pay for effective treatment for headache was higher, signalling dissatisfaction with current treatments.

**Conclusions:**

In Karnataka State, southern India, prevalent headache disorders, especially migraine, give rise to commensurately heavy burdens. Limited access to health care fails to alleviate these. Structured headache services, with their basis in primary care, are the most efficient, effective, affordable and equitable solution. They could be implemented within the health-care infrastructure of India and are likely to be cost-saving. This solution requires political will, itself dependent on awareness.

## Background

Primary headache disorders are not only among the commonest disorders in the world but also among the most burdensome [1, 2], causing pain, substantial disability, lost productive time and, consequentially, very large financial losses [3]. The Global Burden of Disease Study 2010 (GBD2010) reported tension-type headache (TTH) as the second most prevalent disorder worldwide and migraine as the third [1], but migraine far outweighs TTH as a cause of disability. GBD2010 ranked migraine as the seventh highest specific cause of disability in the world [1, 2], but this was an underestimate: data gathered since, and included in the subsequent Global Burden of Disease Study 2013 (GBD2013), elevated it to sixth [4]. Medication-overuse headache (MOH), perhaps best regarded as a complication either of migraine or of TTH, since its occurrence is almost always as a consequence of mismanagement of one or the other, has also entered the top 20 causes of disability in GBD2013 [4].

In the context of India, these facts and statistics take on some significance. First, India has more than 1.2 billion people [5], over 16 % of the world’s population. Second, while headache disorders generally appear to be no less common in this country – at least in Karnataka State – than elsewhere in the world [6], the prevalence of migraine is notably higher than the average reported from other countries [1, 7]. In our first report of this study, the 1-year prevalence of migraine was 25.2 % and of TTH 35.1 %; the prevalence of all headache on ≥15 days/month was 3.0 % [6]. In GBD2010, the estimated worldwide prevalence of migraine was 14.7 % and of TTH 20.8 % [1]. The review of surveys by Stovner et al. estimated the mean global prevalence of all causes of headache on ≥15 days/month at 3.0 % [7]. It is likely, therefore, that very high burdens are attributable to headache in India; but, if so, they are largely disregarded by health services because knowledge of them is not available to inform health policy.

Across the world, knowledge for public-health policy is being gathered by a series of population-based studies supported by *Lifting The Burden* (LTB) [8, 9], a UK-registered non-governmental organisation conducting the Global Campaign against Headache [10] in official relations with the World Health Organization [11]. Methodology has been developed for this purpose [12, 13], focusing on the headache disorders of public-health importance: migraine, TTH, MOH and other causes of headache occurring on ≥15 days/month. One study performed as part of this series was conducted in Karnataka State in southern India. The prevalence data from this study (referred to above) have been published already [6]; our purpose here, again to inform health policy, is to add estimates of burden attributable to these headache disorders, based on enquiries conducted during the survey.

## Methods

The methodology of the study has been published in detail previously [14] and is described only briefly here. The institutional ethics committee of the National Institute for Mental Health and Neuro-Science (NIMHANS) approved the study protocol. Informed consent was obtained from all participants.

It was a cross-sectional survey, conducted during May to November 2009. It sampled urban and rural areas in and around Bangalore: Kempegowdanagara, an urban administrative ward in the city of Bangalore, and Uyamballi and Doddaaladahalli, two large villages located 75–80 Km from Bangalore. Trained interviewers travelled to these communities, selected households through multistage cluster sampling, called at each chosen household, listed all adult members (aged 18–65 years), randomly selected one and interviewed that person using a structured questionnaire. This instrument was an adaptation of the HARDSHIP questionnaire [13], translated into the local language (Kannada) in accordance with LTB’s translation protocol for hybrid documents [15] and validated [14]. The headache screening question (“Have you had headache during the last year?”) and diagnostic questions based on ICHD-II [16] were followed, for those reporting headache, by multiple question sets enquiring into various aspects of burden: symptom burden, lost productive time, health-care utilisation, personal expenditure on health care and willingness to pay for effective care. Any participant reporting more than one headache type was asked to focus only on the one that was subjectively the most bothersome for purposes of diagnosis and burden attribution.

Diagnoses were not made by the interviewers but later, by algorithm applied to the responses to the diagnostic questions. Participants with headache on ≥15 days/month were first identified; among these, any with medication overuse were diagnosed as probable MOH (pMOH), the remainder as “other headache on ≥15 days/month”. The algorithm then applied ICHD-II criteria to all other cases in hierarchical sequence: first for migraine, then for TTH, then for probable migraine and finally for probable TTH. Cases remaining were considered unclassifiable. In the later analysis, migraine and probable migraine were grouped as all-migraine, and TTH and probable TTH as all-TTH.

### Statistics and analyses

Data were entered into a secure database and statistical analyses were performed using EPI INFO [17] and SPSS 15 [18].

We used proportions, 95 % confidence intervals (CIs), means and standard deviations (SDs), medians and quartiles to summarise the distributions of variables, and chi-squared, Student’s *t*-test or ANOVA to test for significance of differences. We used Pearson’s coefficient to test for correlation between continuous variables. We set the level of significance at 5 %.

Headache frequency was reported as headache days/year. Usual intensity was reported by participants on a 3-point categorical score as “not bad”, “quite bad” and “very bad”, which we equated to “mild”, “moderate” and “severe”; these were converted to a numerical rating scale 1–3 and treated as continuous data to derive means (±SD) and medians. We assessed lost productive time as a consequence of headache using the HALT questionnaire [19], enquiring separately into lost paid worktime (days lost from paid employment: questions 1 and 2) and lost household worktime (days lost from home chores: questions 3 and 4). The enquiry period was the preceding 3 months. To estimate the proportion of all productive time lost to each headache type, we assumed there were 240 working days/year. We then calculated the total working days/year available to all participants with each headache type (denominator) as n*240, and used the total reported days lost by them as numerator.

We estimated population-level disability from migraine using the disability weight (DW) from GBD2010 for the ictal state of migraine (0.433) [20]. We calculated mean time spent in the ictal state as a product of mean attack frequency and mean attack duration, and derived from this the proportion of total time that was spent in the ictal state. We multiplied this proportion by the DW to calculate individual disability, and the product by the prevalence of migraine to arrive at population-level disability.

## Results

There were 2,329 participants (1,141 [49.0 %] male, 1,188 [51.0 %] female; mean age 38.0 [±12.7] years; 1,103 [47.4 %] from rural areas, 1,226 [52.6 %] urban). The overall participation rate was 92.6 % (eligible population *n* = 2,514). The distributions of gender, age and habitation in the participating sample, described in detail previously, were comparable to those of the population of Karnataka [14].

Headache in the preceding year was reported by 1,488 participants (63.9 %; 621 male, 867 female), along with attributable symptom burden in terms of frequency and intensity. As reported previously, the age-standardised 1-year prevalence of migraine was 25.2 % and of TTH was 35.1 %; the age-standardised prevalence of all causes of headache on ≥15 days/month was 3.0 % and of pMOH was 1.2 % [6]. Frequency for each of the headache types is presented in Table 1. The majority of participants with migraine had relatively infrequent attacks, but a sizeable minority (227/597; 38.0 %) had ≥3 attacks/month. Overall, migraine was more frequent than TTH, with a median of 2 days/month affected. Migraine was in almost all cases a moderate-to-severe headache, TTH mostly mild-to-moderate; headache on ≥15 days/month, including pMOH, was rated severe by two thirds of those affected (Table 1).

**Table 1:** Symptom burden: headache frequency and intensity by headache type

	Migraine (*n* = 597)	Tension-type headache (*n* = 811)	pMOH (*n* = 28)	Other headache on ≥15 d/m (*n* = 40)
Headache frequency (headache days/year)				
mean ± SD	28 ± 27	17 ± 20	226 ± 59	259 ± 78
median	24	10	216	240
Headache intensity n (%)				
“not bad” (=1)	20 (3.4)	235 (29.0)	2 (7.1)	4 (10.0)
“quite bad” (=2)	346 (58.0)	505 (62.3)	7 (25.0)	9 (22.5)
“very bad” (=3)	231 (38.7)	71 (8.8)	19 (67.9)	27 (67.5)
mean ± SD	2.4 ± 0.5	1.8 ± 0.6	2.6 ± 0.7	2.6 ± 0.6
median	2.0	2.0	3.0	3.0

*n* number of participants with the headache type, *pMOH* probable medication-overuse headache, *d/m* days/month

Lost productive time is shown in Table 2. It should be noted that many median values were zero (*ie*, at least half of people in the category of interest lost no productive time). For the episodic headaches this was so in most categories: for TTH all, and for migraine nearly all. Losses that were spread inconspicuously between paid and household worktime became apparent in total lost productive time. The SDs confirm that distributions were skewed, the usual pattern incorporating a severely affected minority.

**Table 2:** Lost productive time attributed to headache for each headache type, by gender and by urban and rural habitation

	Lost productive time (days per person per 3 months)					
	Total lost productive time		Lost paid worktime		Lost household worktime	
	Mean ± SD	Median	Mean ± SD	Median	Mean ± SD	Median
Migraine						
male (*n* = 212)	2.6 ± 5.6	1	1.3 ± 4.8	0	1.3 ± 2.9	0
female (*n* = 385)	4.0 ± 6.1**	2	1.5 ± 3.6	0	2.5 ± 4.5***	0
rural (*n* = 328)	4.4 ± 6.6	3	1.6 ± 3.8	0	2.8 ± 4.9	1
urban (*n* = 269)	2.3 ± 4.8***	0	1.1 ± 4.4	0	1.2 ± 2.4***	0
Total (*n* = 597)	3.5 ± 6.0	2	1.4 ± 4.1	0	2.1 ± 4.0	0
Tension-type headache						
male (*n* = 388)	1.0 ± 3.1	0	0.4 ± 1.8	0	0.6 ± 1.9	0
female (*n* = 423)	1.4 ± 4.3	0	0.4 ± 2.1	0	1.0 ± 2.6*	0
rural (*n* = 416)	1.5 ± 4.8	0	0.6 ± 2.5	0	1.0 ± 2.8	0
urban (*n* = 395)	0.8 ± 2.1**	0	0.2 ± 1.0**	0	0.6 ± 1.6*	0
Total (*n* = 811)	1.2 ± 3.8	0	0.4 ± 1.9	0	0.8 ± 2.3	0
Probable medication-overuse headache						
male (*n* = 4)	18.3 ± 21.3	9	14.5 ± 24.0	4	3.8 ± 4.8	2.5
female (*n* = 24)	13.1 ± 17.7	7.5	2.4 ± 8.1	0	10.7 ± 11.5	7
rural (*n* = 17)	14.1 ± 22.0	5	6.5 ± 14.7	0	7.6 ± 11.4	5
urban (*n* = 11)	13.5 ± 9.7	10	0.5 ± 1.5	0	13.0 ± 10.0	10
Total (*n* = 28)	13.9 ± 17.9	8	4.1 ± 11.8	0	9.7 ± 11.0	6
Other headache on ≥15 days/month						
male (*n* = 14)	7.3 ± 9.4	2	6.4 ± 9.5	1	0.9 ± 3.5	0
female (*n* = 26)	10.9 ± 9.9	10	4.8 ± 8.4	0	6.0 ± 6.4**	5
rural (*n* = 19)	12.1 ± 11.6	12	6.9 ± 10.0	0	5.1 ± 6.7	3
urban (*n* = 21)	7.4 ± 7.3	5	4.0 ± 7.2	0	3.5 ± 5.4	0
Total (*n* = 40)	9.6 ± 9.7	8	5.4 ± 8.7	0	4.3 ± 6.0	0

*n* number of participants in the category

*** *p* < 0.001; ** *p* < 0.01; * *p* < 0.05

Among participants with migraine, rural dwellers and females lost more time on average than urban dwellers and males, in both cases mostly accounted for by lost household worktime. Similar differences occurred among those with TTH. Much greater losses per person were attributable to the various causes of headache on ≥15 days/month, including pMOH, although again several medians were zero.

Regardless of headache type, lost productivity correlated with headache frequency, both expressed in days/year, albeit only weakly (Pearson correlations: lost paid worktime 0.271; lost household worktime 0.360; total lost productivity 0.402; all *p* < 0.0005).

The estimates of all productive time lost to each headache type are in Table 3. All headache cost 4.3 % of all productive time, females losing more than males (*p* = 0.0155). Migraine caused almost threefold higher losses than TTH (again females more than males, although not significantly [*p* = 0.1021]), but those due to headache on ≥15 days/month, and especially pMOH, were far higher still.

**Table 3:** Estimated proportion (%) of all productive time lost to all headache and each headache type

	Estimated proportion									
	All headache		Migraine		Tension-type headache		pMOH		Other headache on ≥15 d/m	
	*n*	%	*n*	%	*n*	%	*n*	%	*n*	%
Total	1,488	4.3>	597	5.8	811	2.0	28	23.2	40	16.0
male	621	3.0	212	4.3	388	1.7	4	30.5	14	12.2
female	867	5.2	385	6.7	423	2.3	24	21.8	26	18.2

*n* number of participants in the category, *pMOH* probable medication-overuse headache, *d/m* days/month

In expected relation to lost productive time, we made an estimate of the population-level disability from migraine. Participants with migraine reported attacks on 28 days/year on average (Table 1), with a mean duration of 13.1 (±16.9) hours per attack; average time spent in the ictal state as a proportion of all time available to that person was therefore 4.2 % ([13.1/24]*[28/365]*100). Multiplying this by the DW for the ictal state of migraine (0.433) [20], we calculated that each person with migraine carried an average 1.8 % disability burden. Since the prevalence of migraine in the sample was 25.6 % (597/2,329), this disability spread among the whole adult population was 0.46 %. In other words, migraine reduced the functional capacity of the entire adult population by 0.46 %.

We asked about consultations with health-care professionals specifically for headache in the last year. About one quarter of participants responded positively, the proportion depending on headache type. Of those with migraine, 30.3 % had consulted, 84.0 % of these in primary care (Table 4). The proportion was lowest among participants with TTH and far greater among those with headache on ≥15 days/month.

**Table 4:** Consultation with a health-care professional in the last year by headache type

	Migraine (*n* = 597)	Tension-type headache (*n* = 811)	pMOH (*n* = 28)	Other headache on ≥15 d/m (*n* = 40)
Any consultation made (% of n)	30.3	16.6	78.6	72.5
Primary care doctor (%)	84.0	81.6	72.7	51.7
Specialist doctor (%)	13.2	13.6	27.3	41.4
Others (%)	2.8	4.8	0	6.8

*n* number of participants with the headache type, *pMOH* probable medication-overuse headache, *d/m* days/month

Table 5 shows participants’ estimates of their actual expenditure during the last 3 months on health care for headache, including consultations (if any) and medication. The pattern was similar: by far the greatest expenditures were claimed by those with headache on ≥15 days/month, and, among these, by participants with pMOH. Females spent more than males for all specific headache types, and rural expenditure was greater than urban except for pMOH, although, with very wide variations, none of these differences was significant. The table also shows estimated expenditure as a percentage of total quarterly household income, since income must usually constrain what is spent. To make this calculation we multiplied reported monthly income by three. This analysis greatly highlights the expenditure by those with pMOH.

**Table 5:** Participants’ estimates of their actual expenditure during the last 3 months on health care for headache by headache type

Estimated expenditure (INR)	Migraine (*n* = 597)	Tension-type headache (*n* = 811)	pMOH (*n* = 28)	Other headache on ≥15 d/m (*n* = 40)
≤100 (%)	79.7	91.2	21.4	52.5
101–500 (%)	15.2	6.9	53.6	35.0
501–1,000 (%)	2.7	1.2	14.3	2.5
>1,000 (%)	2.3	0.6	10.7	10.0
Mean ± SD	112 ± 307	49 ± 218	532 ± 710	474 ± 1,083
male	71 ± 143	44 ± 232	289 ± 383	517 ± 1,558
female	134 ± 366	54 ± 204	573 ± 748	451 ± 751
urban	103 ± 300	34 ± 177	775 ± 922	330 ± 657
rural	119 ± 314	63 ± 250	375 ± 501	634 ± 1,417
Median	10	5	275	100
% of total household income				
mean ± SD	6.7 ± 49.9	2.4 ± 14.9	32.9 ± 76.9	42.8 ± 172.4
median	0.4	0.1	7.4	1.8

*INR* Indian rupee, *n* number of participants with the headache type, *pMOH* probable medication-overuse headache, *d/m* days/month

Finally, we asked participants how much they would be willing to pay for effective treatment for their headache (meaning that it would no longer bother them), if it were available. This enquiry was intended as an overall measure of burden [12, 13]. Table 6 indicates a gradation: pMOH > other headache on ≥15 days/month > migraine > TTH. It is noticeable (see bottom row) that considerably more would be paid for effective treatments for migraine or TTH than, reportedly, actually had been paid for current treatments.

**Table 6:** Participants’ estimates of their willingness to pay (WTP) for effective treatment for headache, as an overall measure of burden, by headache type

Reported amount willing to pay (INR)	Migraine (*n* = 597)	Tension-type headache (*n* = 811)	pMOH (*n* = 28)	Other headache on ≥15 d/m (*n* = 40)
≤100 (%)	64.8	77.3	28.6	40.0
101–500 (%)	30.7	19.5	57.1	50.0
501–1,000 (%)	3.0	2.0	3.6	7.5
>1,000 (%)	1.5	1.2	10.7	2.5
Mean ± SD	206 ± 452	191 ± 914	435 ± 577	309 ± 376
Median	100	50	200	200
Mean ratio of WTP to actual expenditure	5.8 ± 18.1	8.8 ± 22.3	1.8 ± 3.0	2.4 ± 3.7

*INR* Indian rupee, *n* number of participants with the headache type, *pMOH* probable medication-overuse headache, *d/m* days/month

## Discussion

Our earlier report of this study showed that headache disorders were common in Karnataka State [6]. We observed that these data represented, for the time being, the best information available for the entire country and its more than 1.2 billion people [5]. The age-standardised 1-year prevalence of migraine, at 25.2 %, was well above the global average of 14.7 % [1]. The estimated prevalence of all headache occurring on ≥15 days/month was 3.0 %, equal to the global mean [7, 21], while that of pMOH was 1.2 %, within the range of most national estimates of 1–1.5 % [22].

The importance of these findings may seem self-evident for this very large population, but becomes much highlighted when burden data are added to them. Migraine, so highly prevalent, and the other headache disorders no less common than elsewhere in the world, generate commensurately heavy burdens. Among these are symptom burden – giving rise to health-care demand, disability, personal financial burden, and huge consequential losses in productive time – a large part of which translate into losses from gross domestic product (GDP). We discuss these, although not in this order.

Before doing so, we note the principal limitation inherent in this type of study. Our data were gathered in a cross-sectional survey, by questionnaire, from what we believe were a representative sample, but dependent on recall – in many cases over the preceding 3 months. The error associated with inexact recall has not been established [12], but there is no reason to suppose that it results in over- rather than underestimation.

### Symptom burden

On all measures, migraine was a more burdensome headache than TTH, while the various causes of headache on ≥15 days/month, especially pMOH, were associated with greatest individual burden. In terms of symptoms, obviously the last occurred most frequently (on 245 days/year on average), but they were also, along with migraine, rated severe in intensity. They represent a very high symptom burden for 3 % of the adult population. Migraine occurred on average more than twice a month, which itself is not an unusual finding in the world [7], but 38.0 % of those with this disorder in Karnataka – that is 9.6 % of the adult population – were affected on ≥3 days/month (at least 10 % of days). This has important implications for health care, which we discuss later. Even if no account is taken of TTH, the symptom burden of headache here is heavy.

### Lost productive time

On most measures, females as a group reported greater burden than males, which was largely attributable to higher prevalence [6]. They also tended individually to report greater burden. However, this differential was not clearly seen in lost paid worktime, which almost certainly reflected the local culture of relatively few women being in paid employment. In estimating lost proportions of all productive time, we assumed there were 240 working days per year, which might not have been a fair assumption for household work, but there was no clear basis for any other assumption.

Lost productive time, perhaps especially including lost paid worktime, is one of the major burdens of headache. It has substantial implications not only for those directly affected but also for their dependents and society [12, 13]. In this survey, while the heavy individual burdens attributable to pMOH and other headache on ≥15 days/month are obviously of considerable importance to those affected, societal interest may focus on the 4.3 % of all productive time lost to headache disorders collectively, given that this was the average for the nearly two thirds (63.9 %) of the population who reported headache. This meant the population as a whole aged 18–65 years (effectively the working population) lost 2.7 % (4.3*0.639) of its entire productive time to headache – more than six days a year. This is an enormous loss. Those with migraine (25.2 % of the population) lost 5.8 % of their productive time; they were therefore responsible for a 1.5 % loss from the productive time of the population as a whole – nearly 4 days a year. Lost paid worktime accounted for 40 % of this (Table 2), which might directly detract from GDP.

### Disability

It is of interest to compare this reported lost productivity from migraine (1.5 %) with the disability estimate for the population attributable to those with migraine. This was considerably lower (0.46 %). It is important to recognise that lost productive time as captured by the HALT questionnaire [19] (and MIDAS, from which it derives [23]) is not a measure of disability but of behavioural response to impairment, correlating imprecisely with disability measures while being an important measure in its own right [13]. On the other hand, 5.8 % (the proportion of lost productive time) is only modestly higher than 4.2 % (the average proportion of all available time spent by those with migraine in the ictal state). It is in the nature of migraine that motivation and energy are lost, and that these symptoms, also expected to contribute to lost productivity, may for some time outlast what is described as the ictal state. Important questions therefore arise about the relationship between disability and productivity. In Zambia, with a somewhat lower migraine prevalence of 22.9 % but higher attack frequency (41 days/year) and considerably longer reported mean duration (36 h), the same comparison found the disability estimate (0.98 %) to be similarly smaller than the reported total lost productivity (recalculated using 240 days as the denominator as 3.2 %) [24].

### Health care contact and expenditure

We noted above that 38.0 % of those with migraine (9.6 % of the adult population) were affected on ≥3 days/month. This is often seen as a threshold for prophylactic medication, and therefore for requiring professional health care. In fact, among participants with migraine, not very far short of that proportion (30.3 %) had had contact with a health-care professional (mostly in primary care) at least once in the preceding year. Overall, slightly less than one quarter of participants with headache had seen a health-care professional in the preceding year. Unsurprisingly, much higher proportions (about three quarters) of those with headache on ≥15 days/month had done so, and were much more likely to have progressed to specialist care – yet still they had headache on ≥15 days/month, including pMOH. We were not able to assess how effective the encounters with health-care professionals had been, or the benefits of any health care or advice offered, but it should be recognised that the measured burdens existed despite whatever health care was being provided.

Estimates (by participants) of actual expenditure on health care show that more was spent on migraine than on TTH, but the medians indicate that the majority of those affected, in either case, spent very little indeed. The much higher expenditure by those with pMOH presumably reflected costs of medications, although they also consulted more.

### Willingness to pay

WTP is considered to be an overall measure of burden [12, 13], although it is difficult to ground it. Furthermore, in similar studies in Zambia [24] and Georgia [25], WTP did not correlate with headache type or frequency, or with lost productive time. Clearly its determinants are complex. We are particularly cautious in drawing conclusions related to income, which in the social setting of India may be under-reported, especially by those close to the poverty line, in expectation of higher social security benefits.

What is possible is to make comparisons with participants’ reported actual expenditure. While the latter might be inaccurate, participants would have had those reports in mind when considering WTP. It is therefore informative that the mean ratio of WTP to reported actual expenditure for migraine was close to six and for TTH close to nine. The difference between these probably reflected the much lesser amount expended on TTH, while the low ratios for MOH (1.8) and other headache on ≥15 days/month (2.4) were, even more probably, similarly influenced by the potentially impoverishing expenditures on these disorders. The clear implication of these comparisons is that a level of dissatisfaction existed with current treatments.

### Evidence of unmet need

From all of these findings, the picture emerging in Karnataka is of a high proportion of people in a very large population with heavy symptom burdens attributable to headache, leading to disability and very substantial lost productivity. For whatever reasons, contact with health-care professionals is achieved only by a minority. Apparent dissatisfaction with treatments probably reflects this.

This is a picture of unmet health-care need, because headache disorders are largely treatable [26]. Furthermore, because the lost-productivity (indirect) costs of untreated headache are enormous [3] and treatments can be highly cost-effective [27], there is an expectation that expenditure on treatment would be cost-saving overall [28].

## Conclusions

In Karnataka State, in southern India, a very high prevalence of migraine and levels of TTH, pMOH and other headache on ≥15 days/month similar to global averages give rise to commensurately heavy burdens. Among these are huge losses in productive time, a large part of which translate into losses from GDP. Care must be exercised in extrapolating beyond Karnataka, and we have discussed this issue previously [6]. But for the moment, while this remains the only study of its type in India, it is a fact that the data from it are the best available for all India.

A crucial finding is that the limited access to health care, which the study has demonstrated, fails to alleviate these burdens. Effective treatments exist [26], and could be made available through structured headache services, with their basis in primary care, which are the most efficient, effective, affordable and equitable solution [28]. Their implementation would be with the expectation of substantial cost-saving [27, 28]. The three-tier model proposed by LTB for Europe [29] would readily adapt to the health-care infrastructure of India. This solution requires political will, which itself is dependent on awareness.
